# Auditory voice-perception analysis sensitivity and specificity in the screening of laryngeal disorders

**DOI:** 10.1016/S1808-8694(15)31084-3

**Published:** 2015-10-19

**Authors:** Claudia Alessandra Eckley, Wanderlene Anelli, André de Campos Duprat

**Affiliations:** 1 Doctor in medicine, FCMSCSP. Fellowship in Professional Voice, Thomas Jefferson University - Philadelphia (Assistant Professor); 2 Master's degree in Speech Therapy, Pontificia Universidade Catolica. Head of the Voice Unit, Santa Casa de Sao Paulo; 3 Doctor in medicine, SP Santa Casa. Lecturing Professor, Otorhinolaryngology Department, Santa Casa de Sao Paulo. Otorhinolaryngology Department, Santa Casa de Sao Paulo

**Keywords:** laryngeal cancer, dysphonia, laryngoscopy

## Abstract

Despite the growing experience obtained from the National Pro-Voice Campaigns in screening individuals with laryngeal alterations, we still have not established which would be the best assessment method: speech and hearing screening alone, laryngoscopy alone, or a joint work with physicians and speech therapists doing the screening together.

**Aim:**

the goal of the present study was to assess the sensitivity, specificity, positive and negative predictive values of the auditory voice-perception analysis compared to videolaryngoscopy as a screening method for individuals with laryngo-pharyngeal disorders.

**Materials and Methods:**

We compared the vocal aspects (GRBASI scale, pitch, loudness, CPF and resonance) and videolaryngoscopic from 567 individuals who participated in the National Pro-Voice Campaign 2005 in a tertiary university hospital.

**Results:**

the most frequent laryngeal alteration was laryngo-pharyngeal reflux (LFR) (43.5%), followed by benign lesions (17%) and suspected malignant lesions (1%). The sensitivity of the auditory voice-perception assessment was of 91% for patients with benign lesions and 100% in those with suspected malignant lesions; however, it was only 76% in LFR. Of those tests considered normal, there were vocal alterations in 52%. The positive predictive value was of 71% and the negative was 61%.

**Conclusions:**

Despite its importance, the auditory voice-perception assessment should not be used as a single screening instrument in voice health campaigns.

## INTRODUCTION

Human voice is fascinating and complex. Although the larynx developed phylogenetically to preserve the species, mainly by protecting the airways from aspiration, its phonatory function is just as important in the development of the human race. Voice disorders have become more frequent in a society that uses voice intensely for professional and personal reasons.^1^, ^2^, ^3^, ^4^, ^5^ Brazilian statistics have estimated that about 40% of the economically active population uses voice professionally.^1^, ^2^ Brazil also has one of the highest incidences of laryngeal cancer in the world.^1^ These numbers have motivated the National Voice Campaigns, which have successfully guided people in caring for their voice health and avoiding laryngeal diseases.

There have been many debates about what profile these voice campaigns should have: being purely informative, or including voice screening prior to a laryngoscopic exam, or even a joint evaluation by a physician and a speech therapist. Although the campaigns have an accumulated eight years experience, we have not yet established the most effective manner of assessing the population with laryngopharyngeal complaints.

Otorhinolaryngologists have not traditionally been trained to recognize, quantify, describe and report the rich and subtle features that voice transmits; however, the development and ease of use of optic fibers have enabled detailed and relatively easy evaluations of the pharynx and adjacent structures.^2^, ^3^, ^4^, ^5^ Training of laryngologists increasingly includes associating perceptual and auditory features of voice with laryngoscopic findings based on a wide range of information, which makes it possible to formulate anatomical and functional diagnoses and to suggest therapy that is more aligned with the organ's normal physiology.

The otorhinolaryngologist's work - particularly in laryngology - is not possible without the essential role of speech therapists in voice rehabilitation and training. Multidisciplinary teams are indispensable for fully exercising the sub-specialty of laryngology; however, the scope of each professional needs to be well defined for generating harmonic and well-orchestrated work.

The purpose of this article was to review the sensitivity and specificity of perceptual and auditory testing done by speech therapy teams as a screening process for laryngeal disorders in the general population.

## MATERIAL AND METHOD

The sample included 540 patients who spontaneously sought a tertiary university healthcare facility for an assessment of voice during the National Voice Campaign in 2005; these patients underwent perceptual and auditory voice testing, followed by videolaryngoscopy. The instrument that was used was the official care protocol provided by the Brazilian Academy of Laryngology and Voice, to which were added some extra parameters for perception and auditory analysis. The institution's speech therapists conducted voice testing; this team was composed of speech therapists enrolled in a specialization course on voice, supervised by the teachers of this course. Otorhinolaryngology residents carried out videolaryngoscopy, supervised by their medical teachers in the institution's Laryngology Sector, Otorhinolaryngology Department. The institution's Research Ethics Committee approved this paper. (CEP 124/05).

### Perceptual and Auditory Evaluation

After signing a free informed consent form, patients were interviewed about the reasons for seeking the healthcare unit, the complaints and their duration. During this interview, the speech therapist evaluated the features of connected speech, that is, voice issued during spontaneous speaking. Next, subjects issued the vowels /i/ and /e/ in sustained form, and the /i/ in ascending and descending glissando (continuous scale) for evaluating perception and auditory features. The aforementioned protocol recommends using the GRBASI scale (G=global impression/grade of dysphonia; R=roughness; B=breathiness/soprosity; A=asteny; S=stress; and I=instability);^3^, ^4^ pitch, loudness, resonance and coordination of the pneumophonoarticulatory apparatus (CPFA) were also assessed.^3^, ^4^, ^5^ Pitch (the examiner's perception of voice frequency) was classified as adequate, high or low.^3^, ^4^ Loudness (the examiner's perception of voice volume) was classified as adequate, high or low.^3^, ^4^ The pneumophonoarticulatory apparatus was classified as coordinated or incoordinated.^3^, ^4^

### Videolaryngoscopic Test

The laryngoscopic exam was done using rigid or flexible fibers in all of the patients participating in the Campaign. Topical anesthesia with 10% xylocaine was used in patients that did not tolerate the exam. Rigid or flexible fibers were chosen randomly, except for those with an increased nausea reflex or difficult anatomy for rigid laryngoscopy. Patients that refused the exam or those in which it was not possible to carry out laryngoscopy were excluded from the sample.

### Statistical Analysis

The Epi Info software (version 3.0) for Windows was used for establishing the sensitivity, the specificity, and the positive and negative predictive values of the perceptual and auditory analysis, which were compared with laryngoscopic findings.

## RESULTS

The medical and speech therapy team evaluated 567 subjects in five days of dedicated care. Most of the subjects were adults (88%), of whom 339 were female and 160 were male. There were 68 children, of whom 41 were female and 27 were male.

The initial diagnoses on laryngoscopy were: 219 normal exams (38.6%), 97 exams showing suspected benign lesions of the vocal folds (17%), 4 exams showing suspected malignant lesions (1%), and 247 subjects with signs and symptoms suggesting laryngopharyngeal reflux (43.5%) (Table 1).

**Table 1:** List of 567 subjects according to the initial diagnosis.

Diagnosis	Number of subjects %
Normal Exam	219 38,6
Laryngopharyngeal Reflux	247 43.5
Benign Lesions	97 17.0
Minor Structural Alterations	48 8.4
Nodules	29 5.1
Polyps / Reinke's Edema	12 2.1
Non-Specific Laryngitis	4 0.7
Arching / Presbyphonia	2 0.3
Contact Granuloma	2 0.3
Malignant Lesions	4 0.7
TOTAL	567 100

The sensitivity of the perceptual and auditory evaluation in assessing laryngeal alterations was higher in cases of vocal fold lesions; it was 91% for benign lesions and 100% for malignant lesions. The specificity of this evaluation was 48%, as many subjects that had a normal laryngoscopic exam and no complaints of dysphonia were considered as having voice alterations in the perceptual and auditory evaluation (Table 2).

**Table 2:** Results of the perceptual and auditory evaluation of subjects in the voice-screening program, compared to laryngoscopic findings.

Diagnosis	Subjects		
	Normal voice	Altered voice	Total
Normal Exam	106	113	219
Laryngopharyngeal Reflux	60	187	247
Benign Lesion	9	88	97
Malignant Lesion	0	4	4
TOTAL	173	394	567

The sensitivity for voice assessment was 76% in patients with a suspected diagnosis of laryngopharyngeal reflux (LPR).

In general, the positive predictive value of the perceptual and auditory voice evaluation was 71.2%, and the negative predictive value was 61.3%. The accuracy (number of right answers) of the perceptual and auditory analysis singly was 68%.

## DISCUSSION

The evaluation of individuals with voice complaints requires a trained ear and eyes capable of comparing and interpreting auditory and visual information. Any trained professional may carry out a perceptual and auditory assessment of voice; however, speech therapists are, by vocation and tradition, the best-qualified professionals in their daily activities to undertake this type of evaluation. Multiprofessional teamwork may add knowledge and the ability to approach patients with complex voice complaints that do not correspond to laryngoscopic findings of surfaces.

Care must be taken when using a subjective test to establish the presence or absence of functional lesions in deglutophonatory organs.

In the current study we found that the perceptual and auditory assessment had a relatively low sensitivity for establishing the presence of LPR. A low incidence of organic lesions in the phonatory larynx of these subjects certainly contributed to this finding; most of the alterations were found in the posterior (or respiratory) larynx.^6^, ^7^, ^8^, ^9^, ^10^, ^11^ Laryngeal alterations suggesting LPR were found in 43.5% of patients; this was the prevalent disease in our sample. LPR is considered a risk factor for laryngeal cancer,^6^^,^^7^ and causes significant morbidity;^10^, ^11^ it is, therefore, essential to diagnose and treat this condition as early as possible.

Perceptual and auditory analysis was sensitive and specific for vocal fold lesions; 91% of cases of benign lesions, and all of the cases of malignant lesions were identified using this method. A surprising finding was the large number of false positive cases, individuals with no organic or functional lesions of the larynx or pharynx that were considered as dysphonic in perceptual and auditory testing. Functional conditions might explain a few of these cases; most of them, however, had no voice complaints. Possibly an increased social tolerance of voice alterations in our society may have affected the ability of these individuals to observe voice changes in themselves.^3^^,^^5^^,^^12^, ^13^, ^14^ The reason such individuals sought healthcare was curiosity or complaints of the throat, rather than their voice.

## CONCLUSION

Low positive and negative predictive values in the perceptual and auditory evaluation for assessing and screening laryngopharyngeal alterations, compared with the laryngoscopic exam, suggests that, although perceptual and auditory testing is an essential part of an evaluation of individuals with laryngopharyngeal complaints, it may not be used singly as a screening tool.
